# Origin of the Electroluminescence from Annealed-ZnO/GaN Heterojunction Light-Emitting Diodes

**DOI:** 10.3390/ma8115417

**Published:** 2015-11-16

**Authors:** Kai-Chiang Hsu, Wei-Hua Hsiao, Ching-Ting Lee, Yan-Ting Chen, Day-Shan Liu

**Affiliations:** 1 Institute of Electro-Optical and Materials Science, National Formosa University, Huwei, Yunlin 63201, Taiwan; 10376106@gm.nfu.edu.tw (K.-C.H.); s706802000213@gmail.com (W.-H.H.); a0922639175@gmail.com (Y.-T.C.); 2 Institute of Microelectronics, Department of Electrical Engineering, National Cheng Kung University, Tainan 70101, Taiwan; ctlee@ee.ncku.edu.tw

**Keywords:** electroluminescence, *n*-ZnO/*p*-GaN heterojunction, light-emitting diode, photoluminescence, AES depth profile

## Abstract

This paper addressed the effect of post-annealed treatment on the electroluminescence (EL) of an *n*-ZnO/*p*-GaN heterojunction light-emitting diode (LED). The bluish light emitted from the 450 °C-annealed LED became reddish as the LED annealed at a temperature of 800 °C under vacuum atmosphere. The origins of the light emission for these LEDs annealed at various temperatures were studied using measurements of electrical property, photoluminescence, and Auger electron spectroscopy (AES) depth profiles. A blue-violet emission located at 430 nm was associated with intrinsic transitions between the bandgap of *n*-ZnO and *p*-GaN, the green-yellow emission at 550 nm mainly originating from the deep-level transitions of native defects in the *n*-ZnO and *p*-GaN surfaces, and the red emission at 610 nm emerging from the Ga-O interlayer due to interdiffusion at the *n*-ZnO/*p*-GaN interface. The above-mentioned emissions also supported the EL spectra of LEDs annealed at 700 °C under air, nitrogen, and oxygen atmospheres, respectively.

## 1. Introduction

In recent decades, zinc oxide (ZnO) has attracted significant interest for short-wavelength optoelectronics applications for its wide and direct band gap (*E*_g_~3.37 eV at 300 K) [1,2,3]. Furthermore, ZnO has advantages of excellent resistance to the radiation damage, suitable for the wet-etching process, and large exciton binding energy of 60 meV at room temperature; some ZnO-based optoelectronic applications are expected to be a good substitute for gallium nitride (GaN), another wide band gap (*E*_g_~3.4 eV at 300 K) semiconductor that is the current state-of-the-art approach used for the production of green, blue-ultraviolet, and white light-emitting devices. ZnO has much simpler types of crystal-growth methods, such as sputtering, pulse laser deposition, and hydrothermal methods, resulting in cost-effective approaches for the production of ZnO-based devices [4,5,6]. Among these deposition methods, sputtering technology is a widely used technology for preparing quality and large-sized ZnO film on substrates at a relatively low temperature. However, although there has been some progress made using various growth methods and dopant elements to achieve *p*-ZnO [7,8,9], one current problem that impedes the development of ZnO-based homojunction devices is the lack of reliable and reproducible high quality *p*-type conductivity for ZnO. While *p*-ZnO is difficult to obtain, *p*-GaN which has an almost identical in-plane lattice parameter (lattice mismatch ~1.8%) and wurtzite crystal structure to ZnO film, has been employed to realize an *n–p* heterojunction light emitting diode (LED). Such an LED is made by depositing *n*-ZnO onto the *p*-type GaN epilayer [10,11,12,13]. The luminescent property of *n*-ZnO is known to depend significantly on the crystal structure and content of various defects in the crystal. A short-wavelength band associated with the energy bandgap and a broad long-wavelength band including the involvement of zinc interstitial (Zn_i_), zinc vacancy (V_Zn_), oxygen vacancy (V_O_), and oxygen interstitial (O_i_) defects were generally observed from an undoped *n*-ZnO film [14,15,16]. To accomplish the aim of an *n*-ZnO/*p*-GaN heterojunction LED that emits pure short-wavelength radiation, it is necessary to address and engineer solutions to the above-mentioned visible emission that emerge from defects in *n*-ZnO film, as well as the radiation related to the *n*-ZnO/*p*-GaN interface and *p*-GaN epilayer. Lee *et al*., studied the origin of *n*-ZnO/*p*-GaN heterojunction LEDs annealed under nitrogen and air atmospheres and they obtained a LED emitted blue light after a heterojunction structure was annealed under nitrogen atmosphere [17]. Recently, we also reported a LED that radiated only a near-UV emission when it was constructed from a 450 °C-annealed *n*-ZnO/*p*-GaN heterojunction structure under vacuum atmosphere [18]. Although researchers have endeavored to achieve LEDs that emit a short-wavelength light via thermal annealing of *n*-ZnO/*p*-GaN heterojunction structures, there are few reports that comprehensively discuss the origins of the device emission through the effect of various annealed processes on the heterojunction structures. 

This study measured electroluminescence (EL) spectra as a function of *n*-ZnO/*p*-GaN heterojunction structures annealed at various temperatures and atmospheres. The origins responsible for the evolution of device radiation, that were prepared using *n*-ZnO/*p*-GaN heterojunction structures annealed at various temperatures under vacuum atmosphere, were elucidated with the help of the measurements of the electrical, optical, and material properties of the annealed *n*-ZnO film and an investigation of the annealed *n*-ZnO/*p*-GaN interface. The evolutions of the electrical and optical properties of *n*-ZnO films annealed at an elevated temperature under different atmospheres also were linked to the radiation properties of the resulting *n*-ZnO/*p*-GaN heterojunction LEDs.

## 2. Material Preparation and Experimental Procedure

An undoped ZnO (denoted as *n*-ZnO hereafter) film with a thickness of 100 nm was deposited onto an activated *p*-GaN:Mg epilayer with a hole concentration and carrier mobility of 2.9 × 10^17^ cm^−3^ and 15.5 cm^2^/V·s, respectively, using a radio-frequency (RF) magnetron cosputtering system. A ZnO target (purity, 99.99%) with a diameter of 50 mm was sputtered at room temperature as the working pressure and rf power was adjusted to 1.33 Pa and 50 W, respectively, under a pure argon atmosphere. The pattern of the *n*-ZnO film on the *p*-GaN epilayer was defined using standard lift-off technology. In order to realize an *n*-ZnO/*p*-GaN heterojunction LED, it is important to obtain a quality ZnO film with sufficient carrier concentration. However, since the sputter-deposited *n*-ZnO film exhibited insulated behavior, a post-annealed treatment was employed to apply on the *n*-ZnO film to activate the native donors and to also facilitate crystallinity. Thus, the *n*-ZnO/*p*-GaN heterojunction structure was annealed in a furnace at temperatures ranging from 450 to 800 °C for 30 min under vacuum atmosphere. In addition, as-deposited *n*-ZnO/*p*-GaN heterojunction structures were also annealed under nitrogen, air, and oxygen atmospheres, respectively, at 700 °C for 30 min to provide a comparison to the luminescence property of a vacuum-annealed sample. The Ni/Au (5/50 nm) metal system was deposited onto a *p*-GaN surface and annealed at 500 °C for 10 min under air atmosphere to achieve ohmic contact behavior. Subsequently, a 150 nm-thick transparent indium tin oxide (ITO)-ZnO film [Zn / (Zn + In) = 33 at.%] with an electron concentration and resistivity of 5.3 × 10^20^ cm^−3^ and 5.6 × 10^−4^ Ω·cm, respectively, was deposited onto the patterned *n*-ZnO surface by a RF magnetron cosputtering system, using ZnO and ITO targets. The transparent ITO-ZnO electrode ohmic contact to the *n*-type ZnO was then optimized by a rapid thermal annealing (RTA) process at 400 °C for 5 min in vacuum atmosphere [18]. The contact behavior of the Au/Ni/*p*-GaN and ITO-ZnO/*n*-ZnO systems, respectively, was confirmed by using the transmission-line model (TLM). The Au/Ni/*p*-GaN ohmic contact system exhibited a specific contact resistance of about 10^−2^ Ω·cm^2^, whereas all of the annealed *n*-ZnO contacted to the cosputtered ITO-ZnO electrode showed a specific contact resistance ranging from 10^−3^ to 10^−4^ Ω·cm^2^. Generally, the EL spectrum from an *n*–*p* heterojunction LED was known to be composed of the radiation from the *n*- and *p*-side as well as the *n*–*p* interface. For the *n*-ZnO/*p*-GaN heterojunction LED, in order to extract the radiation emerging only from the *n*-ZnO film without interference from the *p*-GaN epilayer and diffusion at *n*-ZnO/*p*-GaN interface, the hall measurement, photoluminescence, XRD, and SEM data presented in this paper are conducted from the *n*-ZnO film deposited onto the silicon substrates and then annealed under the above-mentioned temperature and atmospheres.

Electrical properties including the carrier concentration, mobility, and resistivity of the annealed *n*-ZnO and cosputtered ITO-ZnO films as well as the activated *p*-GaN epilayer were measured using van der Pauw Hall measurements (Ecopia HMS-5000, Ecopia Anyang, South Korea) at room temperature. The radiative characteristics of the annealed *n*-ZnO films were determined from photoluminescence (PL) spectra measured at room temperature using a He-Cd laser (λ = 325 nm) pumping source. X-ray diffraction (XRD) patterns of the *n*-ZnO film annealed at various temperatures were obtained using a diffractometer (Siemens D-500, Siemens, Munich, Germany) with a Cu Kα radiation source. The corresponded surface morphologies of the annealed *n*-ZnO films were observed using a field emission scanning electron microscope (FE-SEM, JSM-6700F, JEOL, Tokyo, Japan) operated at 3 kV. The evolutions of the annealed *n*-ZnO/*p*-GaN interface were examined by Auger electron spectroscopy (AES) depth profile using a scanning Auger nanoprobe (ULVAC-PHI, PHI 700, ULVAC, Kanagawa, Japan). The current-voltage (I-V) properties of the LEDs fabricated by using annealed *n*-ZnO/*p*-GaN heterojunction structures as well as the ITO-ZnO/*n*-ZnO and Au/Ni/*p*-GaN ohmic contact systems were measured by a semiconductor parameter analyzer (HP4156C, Aglient, Santa Clara, CA, USA). The EL spectra of LEDs fabricated using annealed *n*-ZnO/*p*-GaN heterojunction structures were measured at room temperature under forward injection currents.

## 3. Results and Discussion

**Figure 1:**
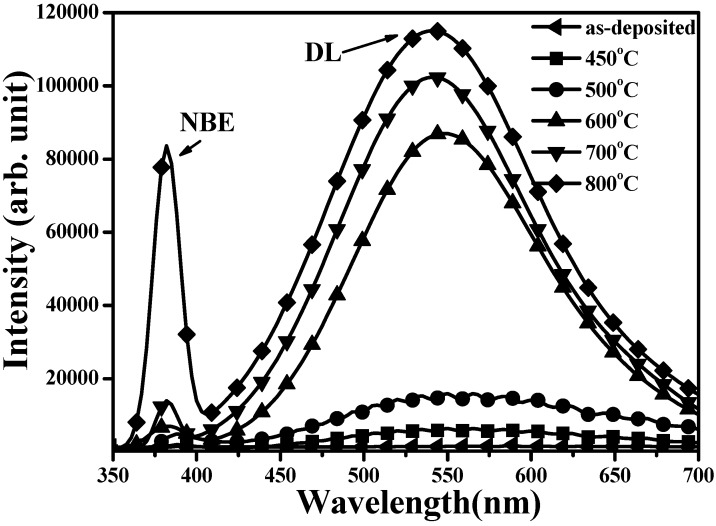
Room temperature photoluminescence (RTPL) spectra of as-deposited *n*-ZnO film and the films annealed at 450, 500, 600, 700, and 800 °C, respectively, under vacuum atmosphere for 30 min.

**Figure 2:**
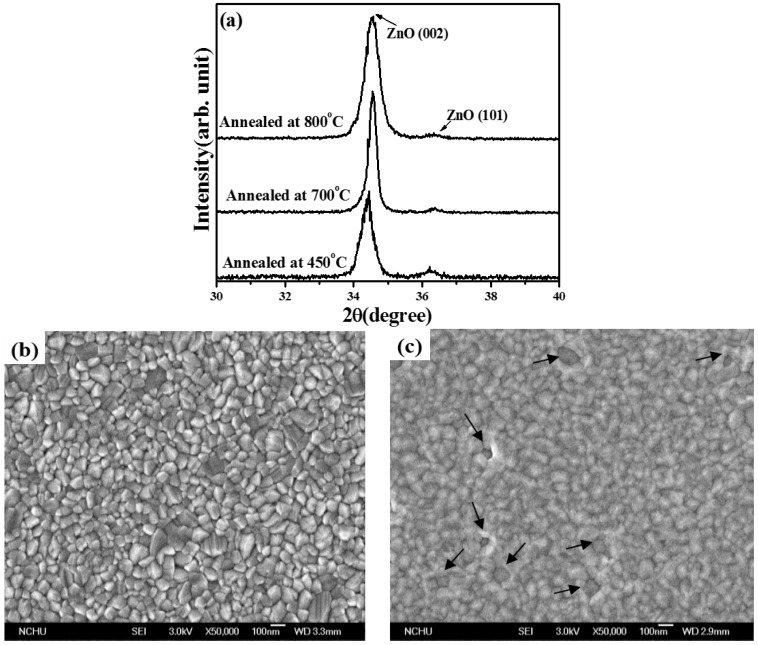
(**a**) X-ray diffraction (XRD) patterns of *n*-ZnO films annealed at 450, 700, and 800 °C, respectively, under vacuum atmosphere for 30 min and surface morphologies of (**b**) 700 °C- and (**c**) 800 °C-annealed *n*-ZnO films.

The electrical properties of *n*-ZnO films annealed at 450, 500, 600, 700, and 800 °C, respectively, under vacuum atmosphere for 30 min are summarized in Table 1. All these films exhibited *n*-type conduction with electron carriers higher than 10^18^ cm^−3^, indicating that large amounts of native donors were activated after the films were annealed under vacuum atmosphere. Carrier concentration and hall mobility both increased with increasing annealing temperature and then decreased as the annealed temperature reached 800 °C. The largest electron concentration of 2.4 × 10^19^ cm^−3^ and the highest carrier mobility of 16.2 cm^2^/V·s were concurrently found from *n*-ZnO annealed at 700 °C under vacuum atmosphere for 30 min. Figure 1 shows the room temperature PL spectra (RTPL) of the as-deposited *n*-ZnO film and the films annealed at various temperatures. Since the sputter-deposited *n*-ZnO film was abundant in non-radiative defects, the emission intensity was too weak to be observed. For the as-deposited film annealed under vacuum atmosphere, two distinct peaks located at near UV (~380 nm) and green (~550 nm) wavelengths were observed from the associated PL spectra. The peak at the short wavelength was denoted as a near-band-edge (NBE) emission that was related to the transition from the energy bandgap of ZnO, whereas the broad emission around the visible wavelengths emerged from the deep-level (DL) emission composed of native defects of V_Zn_, Zn_i_, and V_O_ in the annealed *n*-ZnO film [19,20]. Since atoms at the *n*-ZnO film surface, especially for the oxygen atoms, were in favor of outdiffusion when the film was annealed under vacuum atmosphere at an elevated temperature [21], the peak of the DL emission was close to the peak related to the V_O_ transition, reported at about 580 nm [22]. The slight blue-shift of the DL emission as the annealed temperature increased could be ascribed to the increase in the V_Zn_-related transitions [21]. In addition, both the emission intensity of the NBE and DL were enhanced when the annealed temperature increased as the post-annealed process was favorable for the radiation recombination originating from the improvement in the crystalline structure. This was especially the case for samples annealed at a temperature higher than 600 °C. Therefore, carrier concentrations that were mainly linked to the activation of V_O_ donors and Hall mobility associated with crystallinity increased as the *n*-ZnO film annealed at temperatures increased from 450 to 700 °C, as shown in Table 1. However, although the NBE emission in the *PL* spectrum of the *n*-ZnO film annealed at 800 °C was significantly increased as compared to the DL emission, Hall mobility was lower than in film annealed at 700 °C, implying structural degradation when the *n*-ZnO film annealed at this temperature. XRD patterns of the *n*-ZnO films annealed at 450, 700, and 800 °C, respectively, under vacuum atmosphere for 30 min are shown in Figure 2a. All of these *n*-ZnO films exhibited a hexagonal wurtzite structure with a *c*-axis ZnO (002) growth orientation preference. The evidence of crystallinity as a consequence of the *n*-ZnO film being annealed at a temperature of the 700 °C was seen from the decrease in the full width at half maximum (FWHM) of ZnO (002) in these XRD diffraction patterns. The corresponding crystal size of *n*-ZnO films annealed at 450 and 700 °C, using the Debye-Scherer formula, was about 21.9 and 34.7 nm, respectively. In addition, as quoted from report [23], the shift of the ZnO (002) peak toward a high diffraction angle as compared to bulk ZnO (2θ = 34.42°) was ascribed to the reduction in the *c*-axis lattice constant due to the formation of V_O_ defects for the sample annealed under vacuum atmosphere. Interestingly, although the *n*-ZnO film annealed at 800 °C exhibited the most intense radiation emission, its crystal size apparently shrunk to 17.3 nm, indicating degradation of the crystalline structure. Figure 2b,c shows the surface morphologies of the *n*-ZnO film annealed at 700 °C, and 800 °C, respectively. The surface morphology of the *n*-ZnO film annealed at 700 °C (Figure 2b) was textured with obvious crystal grains. In contrast, the degradation in the crystalline structure of the sample annealed at 800 °C exhibited a surface feature of ambiguous crystal grains. Moreover, the appearance of the porous features as indicated by arrows in Figure 2c was attributed to an excess outdiffusion of the atoms from the surface of the *n*-ZnO film. The increase in grain boundaries was a consequence of the reduction in crystal size and the formation of a porous structure. The grain boundaries obstructed carrier transition, and thereby resulted in the decrease of Hall mobility as listed in Table 1. Accordingly, these films annealed under vacuum atmosphere were both favorable for the growth of crystal size and for enhancement of radiation emission, especially for V_O_-related emission. However, degradation in the crystalline structure and apparent reduction in the crystal size was observed from the sample annealed at 800 °C, where there was a distinct outdiffusion of surface atoms. Furthermore, in terms of the film’s luminescence, the apparent shrinkage in crystal size obtained from the 800 °C-annealed *n*-ZnO film might promote recombination of photo-generated electron-hole pairs due to the size-confinement effect, and therefore the emission intensity increased accordingly [24,25].

**Table 1:** Electrical properties of *n*-ZnO films annealed at 450, 500, 600, 700, and 800 °C, respectively, under vacuum atmosphere for 30 min.

Annealed Temperature	*n* (cm^−3^)	μ (cm^2^/V·s)	ρ (Ω·cm)
450 °C	−1.2 × 10^18^	3.0	3.1
500 °C	−7.3 × 10^18^	7.3	1.2 × 10^−1^
600 °C	−1.6 × 10^19^	9.4	4.3 × 10^−2^
700 °C	−2.4 × 10^19^	16.2	1.6 × 10^−2^
800 °C	−1.7 × 10^19^	12.4	2.4 × 10^−2^

**Figure 3:**
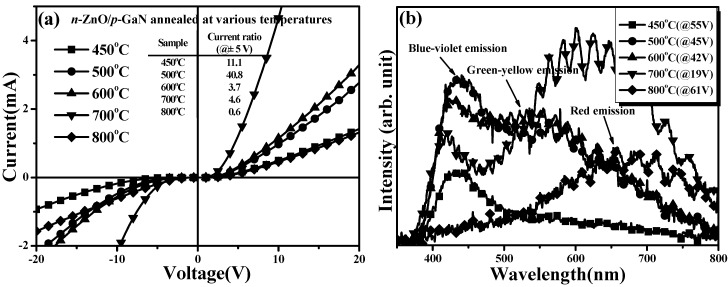
(**a**) Current-voltage (I-V) characteristics of diodes fabricated using *n*-ZnO/*p*-GaN heterojunction structures annealed at temperatures of 450, 500, 600, 700, and 800 °C, respectively, under vacuum atmosphere for 30 min and (**b**) electroluminescence (EL) spectra of these diodes measured under an injection current of 20 mA.

The I-V characteristics of diodes fabricated using *n*-ZnO/*p*-GaN heterojunction structures annealed at temperatures of 450, 500, 600, 700, and 800 °C, respectively, under vacuum atmosphere for 30 min, are shown in Figure 3a. All these diodes exhibited nonlinear behavior with different series resistances and turn-on voltages. Series resistance and turn-on voltage were optimized to 1.65 kΩ and 2.52 V, respectively, for the *n*-ZnO/*p*-GaN heterojunction structure annealed at 700 °C. This was done because the 700 °C-annealed *n*-ZnO film had the lowest resistivity of 1.6 × 10^−2^ Ω·cm and the ITO-ZnO/*n*-ZnO ohmic contact system also exhibited the best contact resistance of 2.9 × 10^−4^ Ω cm^2^. Degradation in the crystalline structure and increase in film resistivity of the 800 °C-annealed *n*-ZnO film resulted in a diode that performed with a high turn-on voltage of 3.24 V and a very high series resistance. The apparent decrease in the current ratio of the 800 °C-annealed sample, measured from the forward turn-on current to reverse leakage current, as shown in the inset table, also implied that there was degradation of the *n*-ZnO/*p*-GaN interface. 

Figure 3b presents the EL spectra of annealed *n*-ZnO/*p*-GaN heterojunction LEDs as a function of the annealed temperatures, measured under an injection current of 20 mA. Only one distinct peak at about 430 nm (denoted as a blue-violet emission in the figure) with a tail extending to the long-wavelengths was obtained from the LED using the 450 °C-annealed *n*-ZnO/*p*-GaN heterojunction structure. For the heterojunction structure annealed at 500 °C, the blue-violet emission was obviously enhanced and another broad peak at about 550 nm (denoted as a green-yellow emission in the figure) appeared in the EL spectrum. The relative intensity of the green-yellow emission from the LED constructed from the 600 °C-annealed *n*-ZnO/*p*-GaN heterojunction structure was almost comparable to that of the blue-violet emission. When the *n*-ZnO/*p*-GaN heterojunction structure was annealed at 700 °C, a broad and intense emission located approximately at 620 nm (denoted as a red emission), became the dominant radiation. However, only one broad and weak emission at about 650 nm, with an ambiguous tail extending to the short-wavelengths, was observed from the LED fabricated using the 800 °C-annealed *n*-ZnO/*p*-GaN heterojunction structure. In general, the electroluminescence of a traditional *p*–*n* heterojunction LED was a factor in the structural quality of the active region and the evolution of the interface. Accordingly, these EL spectra were mainly composed of the radiation from the *p*-GaN side and only a little portion of the emission from the *n*-ZnO side, since the active region of these *n*-ZnO/*p*-GaN heterojunction LEDs was mostly on the *p*-GaN side as conducted from the carrier concentration of *n*-ZnO and *p*-GaN layers. As quoted from reports [17,18,26,27,28], blue-violet emission was attributed to intrinsic transitions of *n*-ZnO film (NBE transition ~393 nm) and *p*-GaN epilayer (shallow donor to the deep Mg-acceptor level transition ~433 nm), as well as the interfacial recombination (~410 nm), whereas green-yellow emission was linked to deep-level defect transitions, such as the V_O_ in the *n*-ZnO and gallium vacancy–related defects (V_Ga_) in the *p*-GaN layers, respectively. In addition, red emission was related to the emission of the Ga–O interlayer originating from the excess oxygen atoms diffused into to the *p*-GaN surface [17,27,29,30]. The peak of the blue-violet emission (~430 nm) from the 450 °C-annealed LED was very close to the radiation from intrinsic transition of the *p*-GaN epilayer as a consequence of the dominant carriers recombination appearing on the *p*-GaN side. The blue-violet emission from the 500 °C-annealed sample was enhanced due to the increase in the annealed temperature and was demonstrated as facilitating the crystallinity of the *n*-ZnO film, which might be in favor of the electron carriers injection as compared to the 450 °C-annealed *n*-ZnO film. Furthermore, this LED also radiated a broad and significant long-green-yellow emission, although the green-yellow emission associated with the V_Ga_-related defects was absent in the *p*-GaN conductive layer [28], indicating that the surface of the *p*-GaN epilayer was damaged with the formation of deep-level defects. Since the vacuum-annealed process was demonstrated to be favorable for the outdiffusion of the oxygen atoms at *n*-ZnO surface, these energetic oxygen atoms at the *p*-GaN surface would greatly enhance the formation of both the V_Ga_ and O_N_ defects at the *p*-GaN surface, which hardly appeared in the *p*-GaN conductive layer. Accordingly, the green-yellow emission associated with the V_Ga_-O_N_ transition was observed from the resulting LED [31,32,33,34]. The noteworthy red emission observed from the LED fabricated using a 700 °C-annealed *n*-ZnO/*p*-GaN heterojunction structure was evidence of the formation of the Ga-O interlayer originating from the oxygen atoms that indiffused into the *p*-GaN surface. Although the single *n*-ZnO film annealed at 700 °C under vacuum atmosphere exhibited the best structural quality as described earlier, the outdiffusion of the oxygen atoms at the *n*-ZnO surface led to degradation of the *n*-ZnO/*p*-GaN interface due to formation of a Ga-O interlayer. 

**Figure 4:**
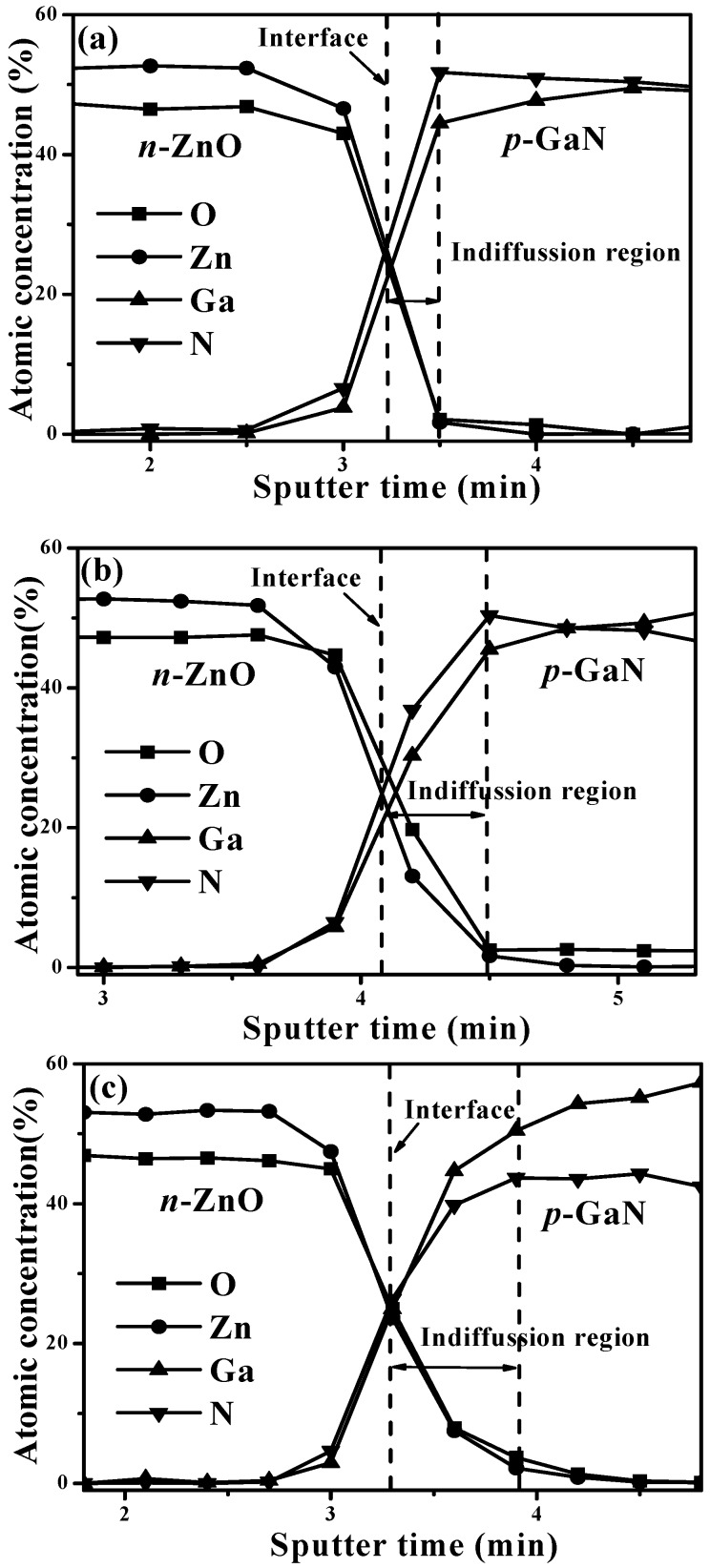
Auger electron spectroscopy (AES) depth profiles of *n*-ZnO/*p*-GaN heterojunction structures annealed at (**a**) 450 °C; (**b**) 700 °C and (**c**) 800 °C, respectively, under vacuum atmosphere for 30 min.

The surface of the *p*-GaN damaged by the oxygen atoms indiffusion led to a decrease in the intrinsic emission from *p*-GaN, and thereby the peak of the blue-violet emission was blue-shifted toward 420 nm which was closer to radiation from interfacial recombination rather than from intrinsic emission from *p*-GaN. In addition, degradation in the crystalline structure due to excessive outdiffusion of atoms in the *n*-ZnO film and enhancement in the formation of the Ga-O interlayer at the *p*-GaN surface might be responsible for the significant decrease in device emission as observed from the 800 °C-annealed LED. The AES depth profiles of the *n*-ZnO/*p*-GaN heterojunction structures annealed at 450 °C, 700 °C, and 800 °C shown in Figure 4a–c, respectively, are presented to study the evolution of the *n*-ZnO/*p*-GaN interface and the origin of the distinct degradation in the intensity of the electroluminescence for LED fabricated using the 800 °C-annealed sample. In these figures, the *n*-ZnO/*p*-GaN interface is determined at half of the Zn atoms as compared to the bulk *n*-ZnO film, while the indiffusion region is denoted as the region from the interface to the saturation of the oxygen atoms in the *p*-GaN epilayer. The atoms in the 700 °C- and 800 °C-annealed samples showed apparent interdiffusion at the *n*-ZnO/*p*-GaN interface as compared to the 450 °C-annealed samples. The indiffusion region that appeared in the *n*-ZnO/*p*-GaN heterojunction structure annealed at 800 °C, which was related to the O and Zn atoms from the *n*-ZnO surface to the *p*-GaN side, was significantly wider than that of the 700 °C-annealed sample. The formation of the Ga-O interlayer originating from the indiffusion of the O atoms at the *p*-GaN side was responsible for the resulting electroluminescence of the LED device being dominated by the red luminescence. In addition, the atomic concentration of N at the *p*-GaN side adjacent to the indiffusion region in the 800 °C-annealed sample (~43%) was markedly lower than the concentration of the Ga (~50.43%), whereas those of the N in the 450 °C- and 700 °C-annealed sample were about 51.7% and 50.3%, respectively, indicating that severe degradation of the *p*-GaN surface had occurred as it was annealed at 800 °C. Since an insufficient number of N atoms was favorable for the increase of the V_N_ donors, the hole carrier concentration at the *p*-GaN surface was thus reduced, and thereby led to significant degradation in the electrical and optical performance of the resulting LED device. Accordingly, although the annealed process on the *n*-ZnO/*p*-GaN heterojunction structures at an elevated temperature under vacuum atmosphere was favorable for enhancing the electroluminescence intensity of the resulting LED, blue-green and greenish light, respectively, was emitted from the 500 °C- and 600 °C-annealed LEDs due to the appearance and enhancement of the green-yellow emission which was related to deep-level defect transitions. By contrast, red emission emerging from the Ga–O interlayer due to indiffusion of oxygen atoms to *p*-GaN became the dominating emission for LED fabricated using 700 °C-annealed *n*-ZnO/*p*-GaN heterojunction structures resulting in the device emitting orange-yellow light. Moreover, significant indiffusion of Zn and O atoms from the *n*-ZnO surface to the *p*-GaN side and degradation in the *p*-GaN layer for the sample annealed at 800 °C weakened the electroluminescence intensity of the resulting LED and thus the device emitted a reddish light.

**Figure 5:**
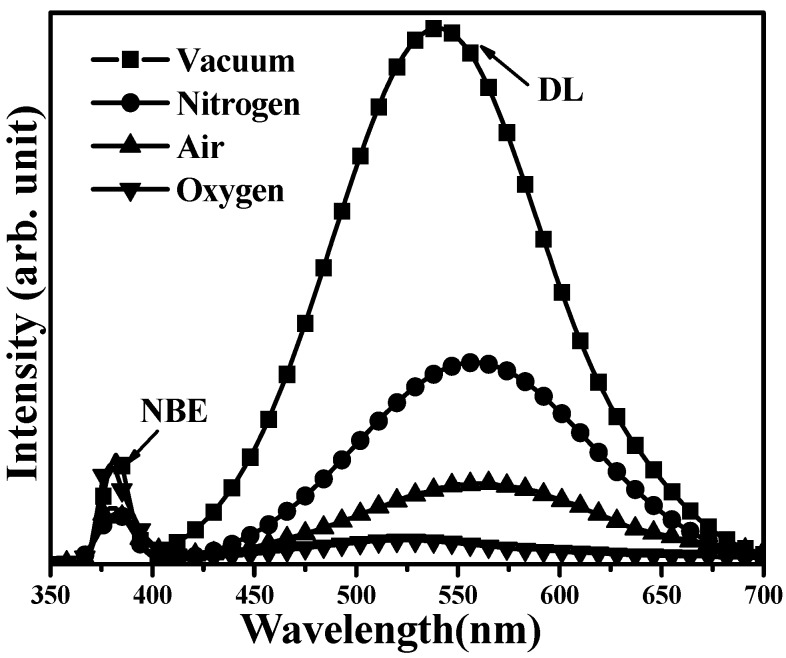
Room temperature PL spectra of *n*-ZnO films annealed at 700 °C under vacuum, nitrogen, air, and oxygen atmospheres, respectively, for 30 min.

**Figure 6:**
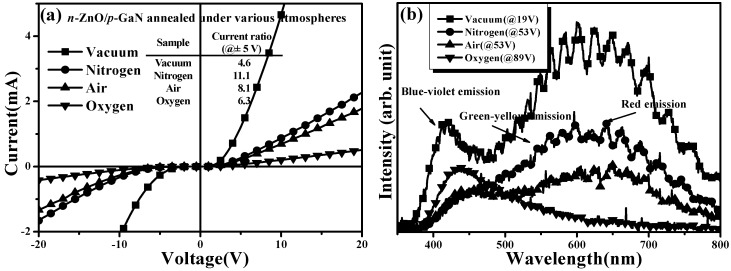
(**a**) I-V characteristics of diodes fabricated using *n*-ZnO/*p*-GaN heterojunction structures annealed under vacuum, nitrogen, air, and oxygen atmospheres, respectively, at 700 °C for 30 min and (**b**) EL spectra of these diodes measured under forward injection current.

Table 2 summarizes the electrical properties of *n*-ZnO films annealed at 700 °C under nitrogen, air, and oxygen atmospheres, respectively (the sample annealed under vacuum atmosphere is also given for comparison). The RTPL spectra of the *n*-ZnO film annealed under various atmospheres at 700 °C for 30 min are presented in Figure 5. The activated electrons in the *n*-ZnO films were suppressed when these gases were introduced into the post-annealed atmosphere. The decrease in the electron carriers also led to the apparent reduction in the DL emission that mainly emerged from the V_O_-related transition as described earlier. Since *n*-ZnO film annealed under the atmosphere incorporating the oxygen atoms was also beneficial for compensation of V_O_ defects [35], the V_O_-related radiation was decreased more effectively for the sample annealed under air atmosphere, and it was almost absent in the RTPL spectrum of the oxygen-annealed *n*-ZnO film. Meanwhile, the electron concentration in *n*-ZnO film annealed under oxygen atmosphere was also minimized to 2.7 × 10^17^ cm^−3^, which was two orders of magnitude lower than the sample annealed under vacuum atmosphere. However, although the V_O_-related defects were compensated by introducing an additive gas during the post-annealed treatment, the crystalline structures of the *n*-ZnO films were thought to be inferior to the sample annealed under vacuum atmosphere as evidence of the marked decrease in carrier mobility. As mentioned in reports [36,37], such degradation in crystalline structure was ascribed to the compression of the crystal size due to the incorporation of ambient atoms in the film surface during thermal annealing, thereby resulting in an increase of the grain boundaries for carrier scattering causing the reduction in the carrier mobility. *I-V* curves of diodes fabricated using *n*-ZnO/*p*-GaN heterojunction structures annealed at 700 °C under vacuum, nitrogen, air, and oxygen atmospheres, respectively, for 30 min are shown in Figure 6a. Turn-on currents of each diode were strongly correlated with the resistivity of these annealed *n*-ZnO films, as listed in Table 2. The higher the resistivity of the *n*-ZnO film, the smaller the turn-on current measured. Figure 6b shows the EL spectra of these LEDs as a function of *n*-ZnO/*p*-GaN heterojunction structures annealed under various atmospheres. The EL spectra of the vacuum-, nitrogen-, and air-annealed LEDs were measured under an injection current of 20 mA, while that of the oxygen-annealed LED was obtained under an injection current of 10 mA. The radiation efficiency of these LEDs fabricated using the *n*-ZnO/*p*-GaN heterojunction structures annealed under nitrogen, air, and oxygen atmospheres, respectively, was inferior to the vacuum-annealed sample. The degradation in device electroluminescence was attributed to their large series resistance and the poor crystalline structure in the *n*-ZnO film. Figure 6b also shows that the relative intensity of the red emission that emerged from the Ga-O interlayer due to the oxygen atoms in the *n*-ZnO surface diffused into the *p*-GaN side was gradually reduced when the annealed atmosphere on *n*-ZnO/*p*-GaN heterojunction structures was incorporated with oxygen gas. A LED emitting bluish light with an absence of red emission was thus achievable when it was fabricated using an *n*-ZnO/*p*-GaN heterojunction structure annealed under oxygen atmosphere.

**Table 2:** Electrical properties of *n*-ZnO films annealed at 700 °C under vacuum, nitrogen, air, and oxygen atmospheres, respectively, for 30 min.

Annealed Atmosphere	*n* (cm^−3^)	μ (cm^2^/V s)	ρ (Ω cm)
Vacuum	−2.4 × 10^19^	16.2	1.6 × 10^−2^
Nitrogen	−1.7 × 10^18^	3.9	9.3 × 10^−1^
Air	−1.2 × 10^18^	2.9	3.2
Oxygen	−2.7 × 10^17^	1.6	7.9

## 4. Conclusions

The origin of electroluminescence from annealed *n*-ZnO/*p*-GaN heterojunction diodes was elucidated with the help of an investigation of the electrical, optical, and material properties of annealed *n*-ZnO films and elemental distribution at the *n*-ZnO/*p*-GaN interface. LED fabricated using an *n*-ZnO/*p*-GaN heterojunction structure annealed at 450 °C under vacuum atmosphere radiated bluish light that was ascribed to blue-violet emission associated with the intrinsic transitions of *n*-ZnO film and *p*-GaN epilayer dominating over the EL spectrum. Although radiation was significantly improved from LEDs constructed from *n*-ZnO/*p*-GaN heterojunction structures that were annealed at temperatures higher than 450 °C, the promotion of deep-level transitions in the *n*-ZnO and *p*-GaN layers, respectively, resulted in an apparent enhancement of green-yellow emission, and as such, 500 °C- and 600 °C-annealed LEDs emitted greenish light. In addition, radiation from LED fabricated using a 700 °C-annealed *n*-ZnO/*p*-GaN heterojunction structure became yellowish as a consequence of the dominating red emission related to the formation of a Ga-O interlayer at the *n*-ZnO/*p*-GaN interface. For an *n*-ZnO/*p*-GaN heterojunction structure annealed at 800 °C, the resulting LED radiated a weak and broad red emission with blue-violet and green-yellow emissions being almost absent due to degradation in both the *n*-ZnO and *p*-GaN surfaces which was confirmed by the crystalline structure of the *n*-ZnO film and the elemental distribution at the *n*-ZnO/*p*-GaN interface. For *n*-ZnO films annealed at 700 °C under nitrogen and air atmospheres, although the formation of V_O_-related defects was effectively suppressed as compared to the vacuum-annealed sample, growth of the crystalline structure was limited. Thus the radiation of the resulting LEDs was obviously inferior to LED constructed from an *n*-ZnO/*p*-GaN heterojunction structure annealed under vacuum atmosphere. Moreover, red and green-yellow emissions related to the oxygen atoms out-diffused from the *n*-ZnO surface to the *p*-GaN side were markedly depressed as the annealed atmosphere incorporated oxygen atoms. LED that radiated almost a blue-violet emission was achievable from an *n*-ZnO/*p*-GaN heterojunction structure annealed at 700 °C under an oxygen atmosphere.
